# Patients’ experience of recurrent/metastatic head and neck squamous cell carcinoma and their perspective on the EORTC QLQ-C30 and QLQ-H&N35 questionnaires: a qualitative study

**DOI:** 10.1186/s41687-018-0060-7

**Published:** 2018-08-01

**Authors:** Arnold Degboe, Sarah L. Knight, Katarina Halling, Andrew Trigg, Tamara Al-Zubeidi, Natalie Aldhouse, Helen Kitchen, Lori Wirth, Simon N. Rogers

**Affiliations:** 1 AstraZeneca, Gaithersburg, MD USA; 2 DRG Abacus, Bicester, UK; 3 AstraZeneca, Gothenburg, Sweden; 4 DRG Abacus, Manchester, UK; 5 Massachusetts General Hospital, Boston, MA USA; 6 Aintree University Hospital, Liverpool, UK

**Keywords:** Qualitative interviews, Head and neck cancer, HNSCC, Oncology, Patient reported outcome, EORTC QLQ-C30, EORTC QLQ-H&N35, Disease conceptual model

## Abstract

**Background:**

Head and neck squamous cell carcinoma (HNSCC) and its associated treatments may affect all aspects of patients’ health-related quality of life (HRQoL). Although the EORTC QLQ-H&N35 is regularly administered to patients with HNSCC, there is a paucity of studies re-assessing the conceptual relevance of this patient-reported outcome (PRO) measure from a patient perspective. Furthermore, the content validity of the EORTC QLQ-C30 has not been widely documented in patients with recurrent and/or metastatic HNSCC. The objectives of this study were to understand patients’ experiences of recurrent/metastatic HNSCC and its treatments, and to evaluate the conceptual relevance and acceptability of the EORTC QLQ-C30 and QLQ-H&N35 from a patient perspective for use in clinical trials.

**Methods:**

A literature review and clinician interviews were conducted to inform in-depth semi-structured telephone interviews with US patients who had received treatment for recurrent and/or metastatic HNSCC in the preceding 12 months. Interview transcripts were analysed thematically using ATLAS.ti v7; patient quotes were coded to identify concepts and themes to develop a conceptual model of HNSCC experience.

**Results:**

Fourteen patients were interviewed (71% male, aged 35–84 years). Patients reported few symptoms pre-diagnosis including neck lump/swelling (*n* = 7/14, 50%) and/or difficulty swallowing (*n* = 3/14, 21%). Treatments generally comprised surgery and chemotherapy and/or radiotherapy. A number of side effects from all treatments were reported. Numbness, difficulty speaking and pain were the most reported side effects of surgery (*n* = 4/8, 50%); weight loss and fatigue were the most reported side effects of chemotherapy and/or radiotherapy (*n* = 8/13, 61%). All side effects negatively impacted patients’ HRQoL. Patients generally found the QLQ-C30 and QLQ H&N35 content to be understandable and conceptually relevant; excessive mucous production and neuropathic symptoms were among the suggested additions.

**Conclusions:**

HNSCC and its diverse symptoms and treatments have a negative impact on many aspects of patients’ lives. A number of reported symptoms including difficulty speaking and swallowing, localised pain and fatigue may be important for treatment benefit evaluation in clinical trials from a patient perspective. The QLQ-C30 and QLQ-H&N35 are generally relevant and suitable for use in clinical trials. However, some items could be amended/added to ensure conceptual comprehensiveness of these measures.

**Electronic supplementary material:**

The online version of this article (10.1186/s41687-018-0060-7) contains supplementary material, which is available to authorized users.

## Background

Head and neck cancer is a collective term that covers malignant tumours arising out of the oral cavity, pharynx, larynx and other anatomic sub-sites. As one of the more common cancers worldwide, head and neck cancer accounts for over 500,000 new cases and nearly 300,000 deaths annually [1]. Squamous cell carcinoma subtype is the most common form of head and neck cancer (over 90%) [2] and arises from various locations in the head and neck region including the nasal and paranasal cavities, nasopharynx, oral cavity, oropharynx, larynx and hypopharynx [3]. Due to the diversity of the affected sub-sites, head and neck squamous cell carcinoma (HNSCC) can manifest in a wide variety of symptoms such as hoarseness, dysphagia (swallowing difficulties), otalgia (earache) and cervical adenopathy (swollen lymph nodes/glands in the neck) [2].

The majority of patients newly diagnosed with HNSCC present with potentially curable localised disease. Treatment options for these patients include surgery, radiation therapy, chemotherapy and biologic therapy. In patients who develop recurrent and/or metastatic disease, palliative treatment with systemic therapy is used in the majority of cases although, immune therapy (e.g. anti-PD-1 antibodies nivolumab and pembrolizumab) are a recently approved treatment option [1].

Patients with HNSCC often experience significant morbidity related to the cancer itself and its treatment, and mortality rates are exceptionally high. Despite recent innovations in surgical techniques, the delivery of radiation, and systemic therapies, the prognosis of patients with locally advanced disease at presentation is poor with a five-year survival of 50%, and a median overall survival of one year in patients with recurrent or metastatic disease [4, 5].

Importantly, symptoms from the tumour (such as pain and difficulty swallowing) and its treatments often result in significant physical impairment (e.g. loss of taste), functional impairment (e.g. difficulty breathing, as well as voice, speech and hearing impairment), and psychosocial problems (e.g. depression, social isolation, and delays returning to work); all of which can have a negative impact on all aspects of patients’ health-related quality of life (HRQoL) [2, 6–8]. Thus, assessing symptoms, changes in physical, functional, and socio-psychosocial impairment, as well as in overall HRQoL, particularly within clinical trials, is necessary not only to evaluate the impact of the disease and its treatments on patients’ lives, but also to make informed decisions on clinical care and rehabilitative services for patients [7–9].

Disease, treatment and site-specific patient-reported outcome (PRO) measures can be used alone, in combination with more general measures (e.g. the Short Form-36) or with clinical assessments such as videofluroscopy for swallowing [10], to assess the impact of HNSCC on patients’ HRQoL and provide an accurate assessment of changes in patient status [8, 9, 11]. Patient experience can also be assessed through qualitative approaches including open- or semi-structured interviews [11]. Notably, patient-reported data can be implemented into clinical practice to improve quality of care for patients with HNSCC [7, 9, 12]. A large number of disease-specific PRO measures are currently available for the assessment of HRQoL in patients with HNSCC [7, 9]. PRO measures vary significantly with respect to their development and validation and no one specific measure has been clearly identified as the gold-standard to assess HRQoL in the HNSCC population [8, 9, 13]. Furthermore, our knowledge on which PRO measures may be most suitable to capture the impact of HNSCC and its treatments on patients in a clinical trial setting is limited. Some work has been conducted in the area of PROs in head and neck clinical trials [14]; however, little research has been carried out to assess specific measures suitability in the field. Factors to consider when selecting such a measure include disease location, treatment, timing of assessment, clinical setting, study purpose, the research question, and the psychometric properties of the measure [7, 11]. However, capturing what is important to patients with the disease, such as the most relevant symptoms and aspects of functioning, should also be considered when selecting a PRO measure.

A number of head and neck cancer PRO measures are commonly used in clinical trials including: the European Organization for Research and Treatment of Cancer Quality of Life Questionnaire Head and Neck Module (EORTC QLQ-H&N35) [15, 16], the Functional Assessment of Cancer Therapy-Head and Neck Subscale (FACT-HN) [17], the University of Washington Head and Neck Cancer Questionnaire (UW-QOL) [18, 19] and the 9-item Disabilities of the Arm, Shoulder, and Hand (Quick DASH-9) [20]. The EORTC QLQ-H&N35 is a 35-item measure designed to assess the HRQoL of patients with HNSCC in conjunction with the general cancer-specific European Organization for Research and Treatment of Cancer Quality of Life Questionnaire (EORTC QLQ-C30); it measures functional status (e.g. physical, role, cognitive, emotional, social), well-being, symptoms and side effects of treatment (both EORTC tools can be found at:: http://groups.eortc.be/qol/eortc-qlq-c30) [7, 8, 11, 15, 16]. The EORTC QLQ-H&N35 is one of the most frequently used PRO measures in the clinical trial setting [7] and is a carefully constructed, thoroughly tested measure with a large body of evidence supporting its psychometric properties [8, 15, 16]. Although the EORTC QLQ-H&N35 is regularly administered in patients with HNSCC [15, 16], there is a paucity of qualitative studies re-assessing the conceptual relevance of this PRO measure to patients in light of the U.S. Food and Drug Administration’s (FDA) PRO Guidance [21]. In addition, the content validity of the EORTC QLQ-C30 has not been widely documented in patients with recurrent and/or metastatic HNSCC, although efforts are underway in a large sample of mixed-diagnosis cancer patients [22]. Similar to other PRO measures, the EORTC QLQ-H&N35 has its strengths and weaknesses, therefore recognising and understanding these in the context of clinical trials is of high relevance. Qualitative research to ensure patient input, for example through patient interviews, may help identify the concepts and questions that are most relevant to patients, and to bridge the gap between QoL research and clinical practice [8]. The opportunity to discuss PRO measures such as the EORTC QLQ-C30 and QLQ-H&N35 at length with patients is very valuable to assess how appropriate these measures are to capture symptom impact and HRQoL in patients with HNSCC.

The aims of this study were: i. to conduct qualitative interviews (consisting of concept elicitation questions and cognitive debriefing of the EORTC QLQ-C30 and QLQ-H&N35) with patients who received treatment for HNSCC in order to explore patients’ experience of HNSCC and its treatments, and understand how these affect their daily activities and functioning, and ii. to evaluate the content validity and relevance of the EORTC QLQ-C30 and QLQ-H&N35 from a patient perspective. The output of data to help answer aim (i) will aid in identifying specific symptoms which may be important to measure in clinical trials.

## Methods

### Literature review

**Fig. 1:**
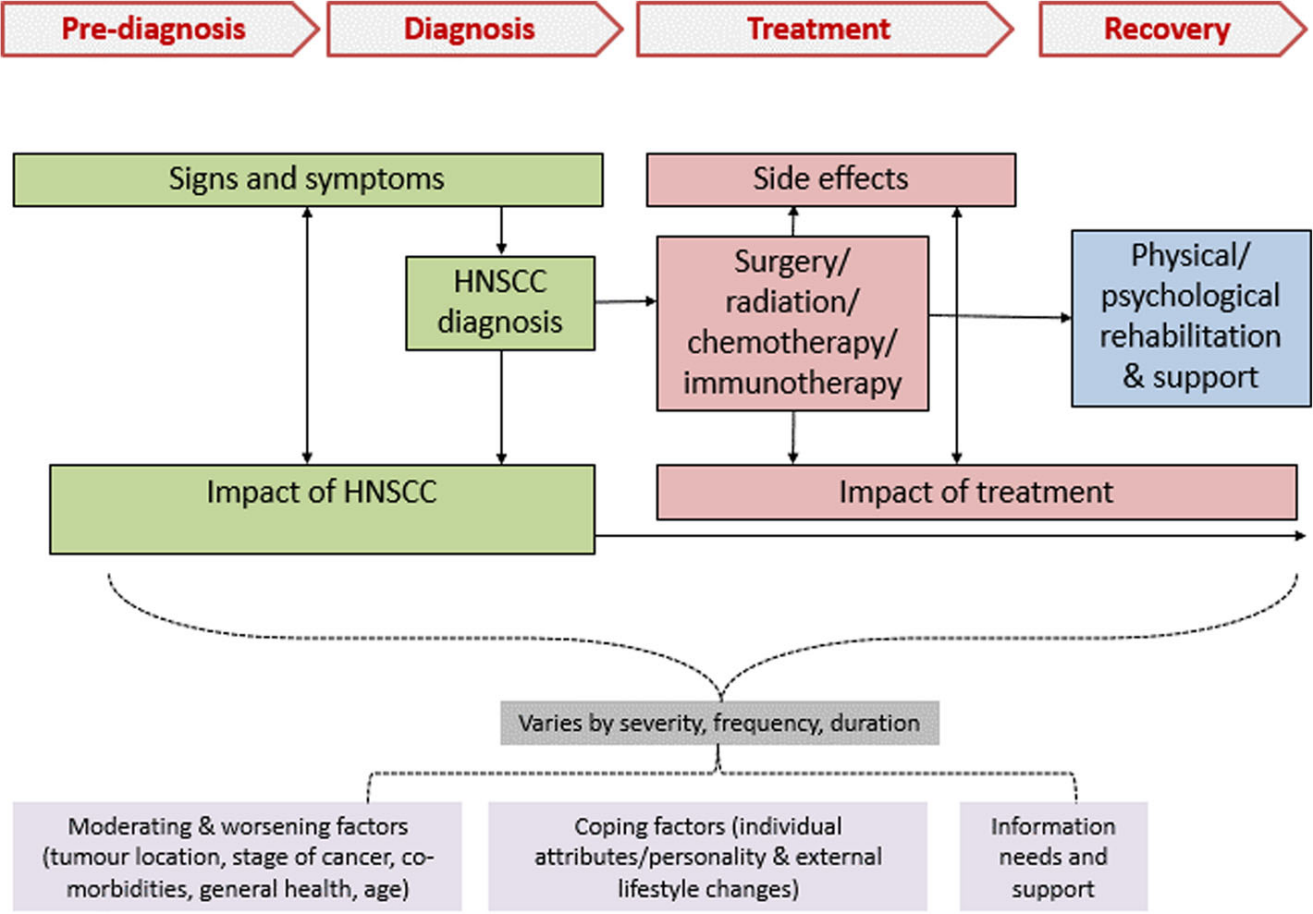
Patients road map in HNSCC. Abbreviations: HNSCC, head and neck squamous cell carcinoma

To assess what is most important and relevant to patients with HNSCC, a qualitative literature search was conducted in the Medline and EMBASE databases to identify published qualitative research in patients with HNSCC (details are not reported in this study [search strategy can be found in Additional file 1: Table S1]). In total, 25 studies were selected and fully reviewed to identify potential measurement concepts, develop a patient road map of HNSCC (Fig. 1), depicting a broad overview of patient experience, which can help inform qualitative interview guides [see Additional file 1 for a reference list of included studies].

### Medical expert interviews

Six qualified healthcare professionals (*n* = 3 from USA, *n* = 3 from EU) with current experience of managing and treating patients with HNSCC (medical oncologists, *n* = 4; surgeon, *n* = 1; specialist research nurse, *n* = 1) took part in a 60-min semi-structured telephone interview to confirm the clinical relevance of the patient road map and identify additional measurement concepts, mentioned to them by their patients, that they deemed important enough to include in the patient road map. The interviews were audio recorded and transcribed to allow in-depth qualitative analysis. Data from these interviews were used to develop a conceptual model.

### Patient interviews

Patients were recruited from two specialist clinical sites in the US (Vanderbilt-Ingram Cancer Centre and Massachusetts General Hospital) and two specialist recruitment agencies applying the eligibility criteria listed in Table 1.

**Table 1:** Eligibility criteria for research interviews

Inclusion criteria	
• Male or female of any race • Aged ≥18 years on the day of the research interview • Confirmed diagnosis of recurrent and/or metastatic HNSCC (oral cavity, oropharynx, hypopharynx, or larynx) • Patient has received treatment for recurrent and/or metastatic HNSCC in the past 12 months • Patient has a WHO Performance Status of 0 or 1^a^ • Patient is willing to take part in a 90-min face-to-face interview (or via Skype or telephone if face-to-face is not possible) • In the opinion of the patient’s physician, patient has the cognitive, reading and linguistic capacities sufficient to allow her/him to actively participate in a 90-min interview • Patient speaks US-English as a first language and can read and write US-English • Patient has personally read, signed and dated a legally effective written informed consent form prior to admission to the study	
Exclusion criteria	
• Patient has a significant psychiatric or physical co-morbid condition that would, in the opinion of the patient’s physician, prevent his/her participation in this study • Patient is currently abusing drugs or alcohol or has done so in 12 months prior to interview • Patient is unwilling or unable to comply with the requirements of the study	

Abbreviations: *HNSCC* head and neck squamous cell carcinoma, *WHO* World Health Organization

^a^Performance status = 0 indicates a patient who is fully active, able to carry on all pre-disease performance without restriction; performance status = 1 indicates a patient who is restricted in physically strenuous activity but ambulatory and able to carry out work of a light or sedentary nature (e.g. light housework, office work)

Ethics approval was obtained from the Institutional Review Boards of Dana-Faber/Harvard Cancer Center for Massachusetts General Hospital and Vanderbilt University. Approval from an Independent Review Board (New England IRB) was also obtained to allow advertisement for additional patients through two specialist recruitment agencies. All patients provided informed consent. All data were anonymised and held confidentially.

A semi-structured interview guide was developed by experienced qualitative researchers and approved by an advisory board of clinical experts. The content of the interview guide was based on information gathered from different sources including the literature review conducted in the disease area (see details in section above) and the qualitative interviews with medical experts in HNSCC.

The semi-structured interview guide was divided into two parts: concept elicitation (which consisted of 6 main questions, and a number of probes for each) and cognitive debriefing (consisting of questions asking about the instructions, recall and understanding for each of the items in the included measures). The concept elicitation section consisted of questions regarding patients’ disease journey (e.g. symptoms experienced), from diagnosis to treatment and its related effects. Open-ended questions were used to avoid biased responses (example questions can be seen in Additional file 1: Table S2).

The cognitive debriefing consisted of an item-by-item assessment, to ask patients the relevance of each item and their understanding of the question and wording for the EORTC QLQ-C30 and QLQ-H&N35, in line with good practice guidelines [23]. The overall instructions, recall period and response options of the measures were also discussed. Patients were asked to complete a paper and pen version of the questionnaire using a ‘think aloud’ technique (i.e. patients vocalised their thoughts as they read each question and selected an answer).

Eligible patients (see Table 1 for eligibility criteria) were invited to participate in a 90-min interview. Telephone interviews were advised by clinical experts to allow patients to remain in their own home between treatments. Due to the nature of the illness affecting vocal cords, speech, and fatigue, patients were able to split the interview over 2–3 separate conversations where necessary; two patients took part in interviews consisting of two different sittings. Patients received an honorarium to compensate for their time involved in the study.

**Fig. 2:**
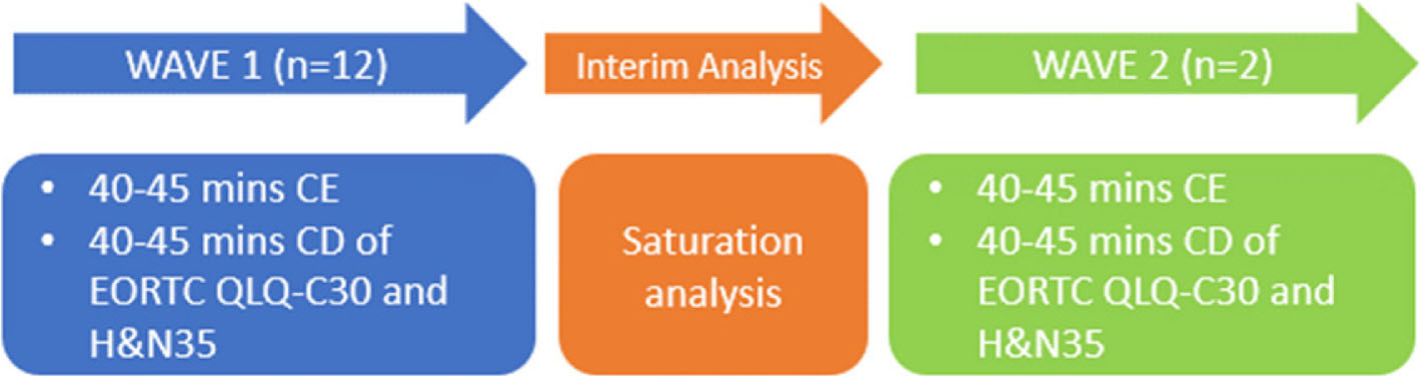
Wave structure of patient interviews. Abbreviations: *CD* cognitive debrief, *CE* concept elicitation; mins, minutes, *EORTC QLQ-C30* European Organisation for Research and Treatment of Cancer, Quality of Life Questionnaire, *EORTC H&N35* European Organisation for Research and Treatment of Cancer, Quality of Life Questionnaire Head and Neck Module. Note: Wave 2 consisted of a smaller number of participants due to conceptual saturation being achieved

Telephone interviews were conducted in English language between December 2014 and January 2017 by experienced qualitative researchers in two waves (Fig. 2).

### Data analysis

All interviews were audio-recorded, transcribed verbatim, and subject to thematic analysis [24] facilitated by ATLAS.ti v7 qualitative data analysis software [25]. Each transcript was read and codes were assigned to sections of text pertaining to common concepts or themes.

### Concept elicitation analysis

Descriptive codes were applied using thematic analysis techniques. Codes were developed into concepts and grouped into domains. Patient-reported concepts were compared and contrasted. A conceptual model was developed using concepts identified from the patient and clinical expert interviews. This consisted of a visual representation of patient experience, including important symptoms and side effects, impacts and potential relationships between them.

### Cognitive debriefing analysis

Concepts identified during the cognitive debriefing section of the interview were tabulated to provide an item-by-item account of patient feedback. Framework coding identified conceptual relevance, item interpretation, and appropriateness of instructions, response scales, response options, and recall period.

### Conceptual saturation

Interim analysis took place after the initial 12 qualitative interviews (wave 1) to determine whether additional interviews were required. Following completion of all the interviews, the transcripts were split into two sets. Concept elicitation segments of the interviews for Set 1 were compared with those for Set 2 to identify any new concepts that may have arisen in Set 2. Conceptual saturation was considered to be attained if no new concepts emerged in Set 2 from the spontaneously elicited concepts, and therefore, no new information would be obtained from additional qualitative data [26]. When a new concept emerged, a retrospective review of previous interviews was conducted to ensure that this concept was not overlooked. All results and data analysis were reviewed by the advisory board of clinical experts, developed specifically for this study, to ensure the results were deemed clinically relevant.

## Results

In total, 14 patients were interviewed; their demographic and clinical characteristics are summarised in Table 2. A diverse range of age, tumour location, time since diagnosis and previous treatments were captured; however, the sample comprised only Caucasian patients, the majority of whom had received higher education. Saturation was not met after review of the first wave of interviews; therefore, further interviews were performed. Following these additional interviews, however, data saturation was deemed to be achieved [presented in Additional file 1: Table S3] because the majority of concepts (especially impact concepts) arose in the first set of interviews. There were some concepts (such as difficulty chewing, ear pain, and headaches) that were outlined in the conceptual model, but described only by clinicians, not patients. For the concepts (such as symptoms of fever, hearing problems, and impotence) that arose only in the second set, nearly all were only mentioned once; highlighting the variability of how HNSCC presents. Interviews allowed in-depth, rich data to be obtained providing a comprehensive insight into the HNSCC patient experience.

**Table 2:** Demographic and clinical characteristics of interviewed patients to be inserted

Characteristic	Total, n (%) *N* = 14
Gender, n (%)	
Male	10 (71)
Female	4 (29)
Age range, years, n (%)^a^	
< 55	2 (14)
55–64	7 (50)
65–74	3 (21)
75–84	2 (14)
Ethnicity, n (%)	
Caucasian or white	14 (100)
Education, n (%)^a^	
Bachelor/graduate degree or higher	10 (71)
High school diploma or equivalent	3 (21)
Missing data	1 (7)
Work status, n (%)	
Full/part- time paid	7 (50)
Retired	4 (29)
Other	3 (21)
Tumour Location, n (%)^a^	
Oral cavity	6 (43)
Larynx	2 (14)
Oropharynx	3 (21)
Hypopharynx	2 (14)
Nasopharynx	1 (7)
Cancer status, n (%)	
Metastatic	10 (71)
Recurrent	4 (29)
Tumour progression during or after treatment	
Yes	4 (29)
No	10 (71)
WHO performance status, n (%)^b^	
0	7 (50)
1	7 (50)
Time since diagnosis, n (%)	
3–6 months	1 (7)
Within last 12 months	2 (14)
1–2 years	7 (50)
> 2 years	4 (29)
Treatment history, n (%)	
Surgery only	1 (7)
Chemotherapy and radiation	6 (43)
Radiation, chemotherapy and surgery	7 (50)
Additional treatments^c^	2 (14)
Comorbidities, n (%)	
Hypertension	3 (21)
Heart disease	2 (14)
Respiratory disease	1 (7)
Cancer (other than H&N)	1 (7)
Other	2 (14)

Abbreviations: *H&N* head and neck. *WHO* World Health Organization

^a^Percentages do not total 100% due to rounding

^b^WHO performance status 0 = fully active, able to carry on all pre-disease performance without restriction; WHO performance status 1 = restricted in physically strenuous activity but ambulatory and able to carry out work of a light or sedentary nature, e.g., light house work, office work)

^c^Additional treatments were: immunotherapy; pembrolizuamb or nivolumab

### Concept elicitation

**Fig. 3:**
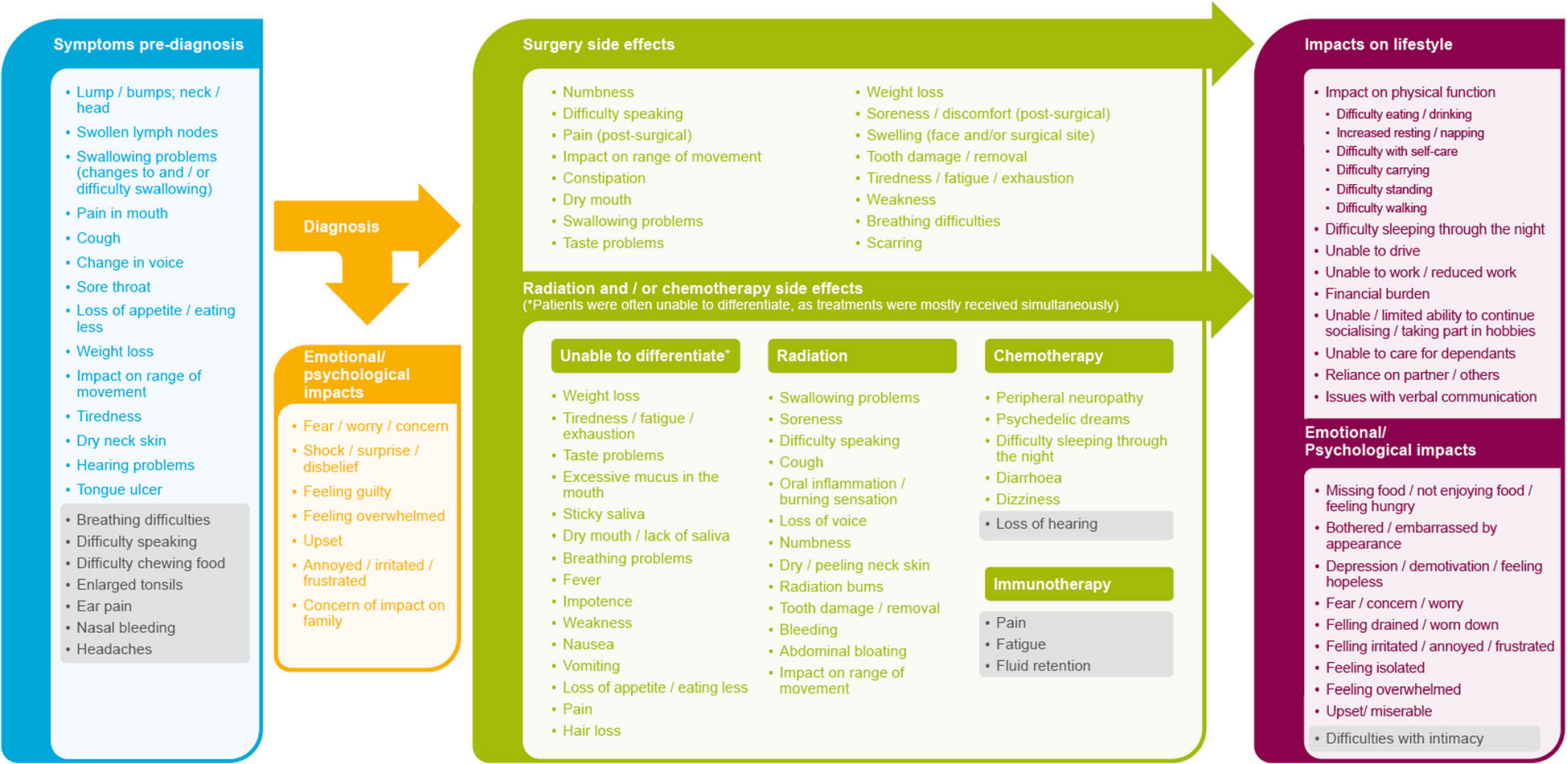
Conceptual model of HNSCC patient experience. The conceptual model was cross-checked with the results of the medical expert interviews. Additional concepts reported by the experts were added to the conceptual model where these were not reported by patients during the concept elicitation interviews (highlighted in the grey boxes). Note: Although immunotherapy is included in the conceptual model, data on side effects experienced is limited by the small number of patients (*n* = 2) receiving this treatment (as part of a clinical trial) and by the fact that they did not attribute any specific side effects to this treatment type

The analysis of the concept elicitation data, combined with input from the qualitative literature and medical experts, led to the development of a conceptual model summarising the experience of patients with HNSCC in terms of measurement concepts and appropriate overarching themes. The conceptual model can be used to guide potential areas suitable for patient-reported measurement, and confirm content validity of patient-reported questionnaires. As demonstrated in Fig. 3, the patient experience of HNSCC could be segmented into pre-diagnosis signs and symptoms, emotional and psychological impacts of receiving a diagnosis, side effects due to various treatments, and overall impact on patients’ life in addition to emotional and psychological wellbeing.

During the concept elicitation part of the interview, patients were asked to describe their experience of HNSCC, including what symptoms they experienced, what lead them to get medical input, and how their diagnosis affected them. Prior to diagnosis, the most commonly reported signs or symptoms of HNSCC were lumps or swelling in the neck (*n* = 7, 50%) or difficulty swallowing (*n* = 3, 21%):

*“Basically when I was first diagnosed I had a lump on my neck, on my right hand side of my neck. I noticed it for maybe two months, it did not bother me, did not hurt.*” (01–06-M-57)

*“I first had a … difference in my swallow.”* (01–04-M-59)

These problems negatively impacted patients ability to eat/drink and speak. Upon receiving a diagnosis of HNSCC, patients reported feeling shocked, surprised and/or disbelief. One patient expressed great frustration at the length of time it took for his (or her) diagnosis to be reached. Another described feelings of guilt for not having his (or her) symptom (lump on tongue) evaluated sooner; the same patient also reported feeling overwhelmed after receiving his (or her) diagnosis. An overview of the signs and symptoms experienced by patients prior to treatment are outlined in Table 3.

**Table 3:** Overview of signs and symptoms experienced by patients prior to treatment

Symptom	Reported			Example quote
	S	P	Total	
Lumps; head/neck	6		6	• *“Basically when I was first diagnosed I had a lump on my neck, on my right hand side of my neck. I noticed it for maybe two months, it did not bother me, did not hurt.”* (01–06-M-57)
Difficulty/difference in swallowing	3		3	• *“I first had a… noticed a difference in my swallow... it was something that was different.”* (01–04-M-59)
Pain	1	1	2	• *”.. my mouth it would hurt and I knew there was something wrong.”* (01–07-F-66)
Swollen lymph nodes (neck)	2		2	• “…nothing else other than the visible swelling of the lymph nodes and no pain, nothing else.” (01–03-M-55)
Tongue ulcer	1		1	• *“Initially I had a sore on my tongue that didn’t heal so I was sent to an oral surgeon and then an ear, nose and throat doctor.”* (01–14-F-61)
Weight loss		2	2	• *“I started losing weight but not a lot at that time... I’m watching my weight go down and going okay but it wasn’t going down that much.”* (01–07-F-66)
Change in voice		1	1	• *“That would be going back to like a raspy throat like what I had prior to being diagnosed, I had this… It would seem like my mouth was raspy and noticeably kind of different pitched.”* (01–09-M-66)
Cough	1		1	• “I *started to get a little more noticeable that I kept clearing my throat and coughing a little bit and so I decided to go to the doctor’s thinking maybe there was an infection or something of that nature.”* (01–09-M-66)
Dry/peeling skin on the neck		1	1	• *“It felt like when you go swimming and your skin dries out, that’s how my neck was feeling, every two or three days or something like that.”* (01–10-M-35)
Eating less		1	1	• *“I told him I used to be somewhat hungry all three times of the day, but it’s shifted now to only two times of the day.”* (01–10-M-35)
Hearing problems	1		1	• *“I was having hearing problems…so I went to an ENT. He ran some tests on me and then he said we have a problem, we’re going to drill up and do a biopsy, and that’s when they found I have a nasopharynx tumour.”* (01–13-F-62)
Impact on range of movement		1	1	• *“So my hands were throwing to a certain direction, but my eyes or my face wouldn’t turn in that direction, just because of the neck drying out, slowing up, stiffen up.”* (01–10-M-35)
Tiredness		1	1	• *“I said I felt like I was getting enough sleep, but I still feel a little tired.”* (01–10-M-35)

Abbreviations: *P* reported when prompted, *S* reported spontaneously

Patients were also asked about the treatment that they had received for their HNSCC. Thirteen patients received both chemotherapy and radiation treatment, seven of which also received surgery; the remaining patient had only received surgical treatment (Table 2). Due to the range of treatments received, the specific source of various side effects and treatment-related impacts was difficult for patients to attribute. However, since surgery was often an isolated event with regards to other treatments, the side effects or complications of this were more readily discernible. All patients who underwent surgery in combination with other treatments or alone (*n* = 8) experienced some side effects resulting from their surgery; numbness around the site of surgery (mouth, cheek, tongue and neck; *n* = 4, 50%), post-surgical pain (*n* = 4, 50%) and difficulty speaking (*n* = 4, 50%) were the most frequently reported (Table 4).

*“I had 20 staples in my neck and I woke up in a lot of pain … I couldn’t talk. My friends would call and family to see how I was and it was very difficult talking on the phone initially” (01–14-F-61)*

**Table 4:** Side effects of surgery and chemotherapy and/or radiotherapy in patients with HNSCC

Surgery				Chemotherapy and/or radiotherapy			
Side effect	Reported by (*N* = 8)			Side effect	Reported by (*N* = 13)		
	S	P	Total		S	P	Total
Numbness	1	3	4	Weight loss	1	7	8
Difficulty speaking		4	4	Tiredness/fatigue/exhaustion	3	5	8
Pain	2	2	4	Taste problems		6	6
Swelling	1	2	3	Pain	3	3	6
Dry mouth		2	2	Dry mouth/lack of saliva		5	5
Soreness/discomfort		2	2	Nausea		5	5
Weakness		2	2	Hair loss	1	1	2
Tired/fatigue/exhaustion		2	2	Vomiting	2	1	3
Swallowing problems	1	1	2	Weakness		3	3
Constipation		1	1	Breathing problems		2	2
Impact on range of movement		1	1	Excessive mucous		2	2
Taste problems		1	1	Loss of appetite/eating less		2	2
Tooth damage/removal		1	1	Sticky saliva		2	2
Weight loss		1	1	Fever		1	1
Scarring	1		1	Impotence		1	1

Abbreviations: *N* total number of patients reporting side effects, *P* reported when prompted, *S* reported spontaneously

Side effects resulting from chemotherapy and/or radiation included tiredness/fatigue/exhaustion (*n* = 8, 61%), taste problems (*n* = 6, 46%), nausea (*n* = 5, 38%) and loss of appetite (*n* = 2, 15%), all of which may have contributed towards the significant weight loss experienced by many patients (*n* = 8, 61%). Patients also reported various oral side effects including alterations in their sense of taste and changes to saliva production e.g. sticky saliva/excessive mucous (Table 4). Pain was reported mainly in relation to pain at radiation sites; however, one patient reported experiencing pain after chemotherapy:

*“After it was done I felt a little bit of pain…it was pain, but I guess it didn’t last too long, but it was still there.”* (01–10-M-35)

More readily distinguishable side effects deemed specific to radiation included soreness (*n* = 5, 38%), burns (*n* = 5, 38%) and dry skin at the site of radiation (*n* = 4, 31%):

“*The radiation you do have… your neck gets burned almost like a sunburn*” (01–03-M-55)

Impacts on emotional and psychological wellbeing throughout the course of patients’ HNSCC were also readily discussed. Patients reported feeling fearful for the future and their families, and feeling depressed and upset. The four patients who had experienced recurrence described feeling helpless, overwhelmed, and scared upon receipt of this news.

*“I felt really very bad when I found out the cancer had come back, just hopeless” (01–15-F-32)*

In the patient sample, only two patients had received immunotherapy as part of a clinical trial and they did not attribute any specific side effects to this treatment type.

### Cognitive debriefing

During the second part of the interview, patients were asked to complete the EORTC QLQ-C30 and QLQ-H&N35 aloud, and provide their thoughts. The majority of patients (*n* = 12, 86%) reported that the EORTC QLQ-C30 and QLQ-H&N35 were suitable and easy to complete. The key findings from the cognitive debriefing interviews are presented in Table 5 and elaborated on subsequently.

**Table 5:** Summary of cognitive debriefing findings

Consideration	Findings	Example quotes
Conceptual relevance	• All items deemed relevant by patients • Most key symptom/side effect and impact concepts assessed, although some are missing (e.g. neuropathic symptoms, excessive mucus production)	• *“I: Is there anything important you feel that’s missing from either of the questionnaires?* *01–06-M-57: No.”* • *“You could probably ask about there wasn’t anything really on here about nerves, neuropathy, whether your feet or hands or fingers are still normal or tingly, numb, anything like that, because I think that must happen quite a bit with chemo…” (01–04-M-59)*
Interpretation and understanding	• Instructions well understood and consistently followed • Items generally well understood and interpreted consistently; problematic items included those assessing a ‘long’ or ‘short’ walk, and “sticky saliva”; mainly associated with the definitions of what these consisted of	• *“They are asking me to do a response to the questions by circling the number that applies best to my condition and my situation.” (01–05-M-57)* • *“That I just think it’s confusing... I would say a distance or even a timeframe…maybe you can say a 20-min walk or a one mile walk.” (01–10-M-35)*
Response scale and options	• Largely considered appropriate • Patients with feeding tubes at the time of study completion had difficulty responding to items related to eating and swallowing	• *“I: The response options; do you think they’re suitable? The four response options?* 01–14-F-61: Yes.” • *“I: That’s quite an important point you’ve brought up there, obviously some of these are not relevant to you. Do you feel that there should be an answer that says ‘not relevant’?* *Yes.” (01–12-M-70)*
Recall period	• Recall employed for Items 1–5 varied, due to no specified recall period • The recall period of the remaining items was easily understood but not always adhered to throughout	• *“I: Again, the recall period, what recall period were you thinking of?* *01–15-F-32: Right now these are last week as well.”* • *“The only comment I made at the beginning, being clear about the timeframe one through five, and once you get past one through five you understand. But I think that would be… you could go through the first five questions and answer them with the wrong timeframe.” (01–03-M-55)*

### Conceptual relevance

Every item of the EORTC QLQ-C30 and QLQ-H&N35 was described as relevant by at least one patient regarding their individual experience. While the majority (*n* = 12, 86%) of patients felt that the questionnaires covered the most important aspects of their disease experience, two patients suggested adding items relating to side effects they had experienced: excessive mucous production and neuropathic symptoms.

*“You could probably ask about … nerves, neuropathy, whether your feet or hands or fingers are still normal or tingly, numb, anything like that, because I think that must happen quite a bit with chemo…”* (01–04-M-59)

*“Yes, you need to expand on the mucous thing … it is very discomforting”* (01–03-M-55)

### Interpretation and understanding

Patients were able to follow the instructions as intended when completing the measure, although one patient suggested that the instructions for the EORTC QLQ-C30 items could be improved by specifying that the questionnaire is concerned with aspects of health relating to cancer and its treatment, rather than ‘you and your health’. Another patient was unsure if they should have responded to the EORTC QLQ-H&N35 items specifically in reference to their treatment side effects.

*“They are asking me to do a response to the questions by circling the number that applies best to my condition and my situation.”* (01–05-M-57)

*“…if it says something that closely relates to you … we are interested about your treatment for cancer or your treatment about a serious severe illness, then you know that pinpoints it. Because if you think about it health could be anything.”* (01–10-M-35)

*“But what I don’t understand is there might be some things that have happened that are not related to the surgery and so [do I] answer yes for those, or...?”* (01–11-M-62)

Whilst the majority of items were understood consistently by all patients, some issues were apparent regarding the wording of five items. The wording of Item 2 (‘Do you have any trouble taking a long walk?’) was considered unclear by two patients as they felt that the item needed to be more specific as to what a ‘long walk’ is. A similar opinion was expressed for Item 3 (‘Do you have any trouble taking a short walk outside of the house?’). Patients interpretation of a ‘long’ and ‘short’ walk varied; ‘long’ could be considered ‘more than a mile’ or ‘over 5 minutes’, while short could be considered ‘a block or so’ or ‘two doors down’.

*“It’s confusing … I would say a distance or even a timeframe … maybe you can say [a long walk is] a 20-minute walk or a one mile walk*.” (01–10-M-35)

Regarding Item 36 (‘Have you had problems swallowing pureed food?’), one patient was unsure what constituted ‘pureed’ food.

*“I don’t know what the difference is between solid food and pureed food. Pureed food supposed to be like apple sauce or something?”* (01–10-M-35)

Three patients found the term ‘sticky saliva’ in Item 42 (‘Have you had sticky saliva?’) unclear.

*“Sticky saliva, I don’t know what sticky saliva is, is that just mucous or sticky saliva, what’s the difference?”* (01–04-M-59)

Furthermore, three patients struggled to correctly understand Item 62 (‘Have you taken any nutritional supplements [excluding vitamins]?’); two read the question as ‘including vitamins,’ rather than excluding vitamins and one did not know whether to include nutritional food within this.

### Response scale and options

Overall, patients found the response options for the QLQ-C30 and QLQ-H&N35 understandable and relevant. However, two patients struggled with the response options and reported trouble interpreting them for their symptoms which included diarrhoea and vomiting (Items 15–17).

*“Yes, I think based on the questions they are asking I think they are appropriate.” (01–05-M-57)*

*“Okay have you had diarrhoea? Well I’ve had loose stools a couple of times but I haven’t had it constantly. You know, so how do you answer it? …I mean because you know sometimes things happen to you but not all the time.” (01–07-F-66)*

Patients with an assisted feeding tube (*n* = 3, 21%) felt that none of the response options were applicable for several items related to appetite, eating and swallowing food (Items 13, 36–38, 49–52). One considered the addition of a ‘Not relevant/Not applicable’ option to be more appropriate.

*“Right now I can’t have any food, it’s in my feeding tube … that’s another non-answer because I don’t have meals.” (01–13-F-62)*

### Recall period

Generally, the recall period was understood and used correctly; however, two patients used it incorrectly, and three patients suggested changes to help clarify the recall for Items 1–5 of the EORTC QLQ-C30, which does not specify a recall period. Patients varied greatly in the timeframes they considered when answering these questions, from ‘now’ to ‘the last year and a half’.

*“…the only thing I would probably make clearer is in your current timeframe maybe, those [first] five questions, so you know its present.” (01–06-M-57)*

For the remaining items, the minority of patients (*n* = 5, 36%) who did not use ‘the past week’ recall period were often thinking back to their current or recent treatment period. This is unlikely to be an issue when the measure is issued during a clinical trial.

*“I’m thinking of from treatment to now.” (01–07-F-66)*

*“I was averaging over this treatment.” (01–12-M-70)*

## Discussion

To our knowledge, this appears to be a novel qualitative study aimed at exploring patients’ experiences of recurrent/metastatic HNSCC and its treatments, and to evaluate the conceptual relevance and acceptability of the EORTC QLQ-C30 and QLQ-H&N35 from a patient perspective. Other studies have assessed the use of these PRO measures, including assessing the QLQ-C30 for content validity [22, 27–30]; however, none of these studies appear to have debriefed the PRO measures following an approach similar to that used in this study [31]. Patients’ experiences were depicted in the form of a conceptual model sectioned into pre-diagnosis signs and symptoms, emotional and psychological impact of receiving a diagnosis, side effects due to various treatments, and overall impact on patients’ life and emotional and psychological wellbeing. This conceptual model highlights the impacts and burden of HNSCC on patients some of which may be suitable to capture through patient self-report.

Several PRO measures specifically designed for use in patients with HNSCC are currently available, with the EORTC QLQ-C30 and QLQ-H&N35 being the most frequently used [7]. The wide and frequent use of these PRO measures together with the changes in the treatments for HNSCC over time justify a robust examination of their strengths and weaknesses and potential adjustments to ensure accurate assessment. During the development and initial validation of these European Organization For Research And Treatment Of Cancer (EORTC) measures, limited patient input and feedback was sought [15, 16]. Unlike the initial validations, this study incorporated cognitive interviewing techniques to debrief both questionnaires, enabling a more thorough understanding of their relevance and acceptability. Furthermore, modern advances in therapy in addition to societal changes since the questionnaires’ development also justify an updated debriefing. It is important to note that an updated version of the EORTC QLQ-H&N35 is under development (the QLQ-H&N43) [32–34]; however, the final version was not available for consideration in this study. The update of this PRO measure was felt advisable given the deficits in the original EORTC head and neck cancer module in relation to targeted and/or multimodal therapy [32, 34]. The preliminary revised module includes 17 additional items (a number of which were identified in this study, such as “tingling or numbness in hands or feet”) and one altered item; nine items of the H&N35 were removed [32]. Prior to use in clinical trial, further debriefing through in-depth cognitive interviewing will be warranted once the update is finalised.

Results from this study suggest that all items were deemed relevant by patients, however, neuropathic symptoms and excessive mucus production were cited as ‘missing’ concepts. The symptoms and impacts reported by patients during this study may be used to inform the selection and prioritisation of endpoints during clinical trials. From the insight gained from the patients during this study, it would appear that patients identified difficulty speaking/slurred speech, difficulty swallowing (and therefore eating/drinking), localised pain, and fatigue as the most significant physical impacts.

Although some new concepts arose in the second set of data during qualitative analysis, they were usually unique to the specific patient who reported them; this is unsurprising due to the heterogeneity of tumour location and disease experienced by the patient sample. Therefore, saturation was deemed to be obtained. The appearance of new concepts is not uncommon in studies such as this, and it is important to use scientific judgement, including knowledge in the field, as well as consultation with experts to determine how important any new concept is (e.g. is it minor, technically unrelated, or an outlier) [35]. For this study, this approach was followed and further judgement from the research team and the advisory board of clinical experts determined that additional data collection was not warranted.

The omission of elicited concepts (see Fig. 3) in the EORTC questionnaires is notable, although a previous review reports their content coverage as the widest of nine HNSCC-specific questionnaires [13]. Despite this, it is recognised that no single measure is ideal for all scenarios and can fully assess every possible issue; the specific context should be considered. For example, a recent paper authored by several members of the FDA’s Center for Drug Evaluation and Research proposed a focus on the measurement of symptomatic adverse events, physical function, and disease-related symptoms [36]. Any additional treatment-related side effects of interest, but not covered by the questionnaire, could be assessed through selecting appropriate items from a bank such as the Patient-Reported Outcomes version of the Common Terminology Criteria for Adverse Events (PRO-CTCAE) (e.g. its ‘numbness and tingling’ item to capture neuropathic symptoms) [37]. Alternatively, PRO measures such as the Patient Concerns Inventory incorporate an optional free-text item [38].

The questionnaires were generally well understood. Special care should be taken when administering the EORTC QLQ-C30 and QLQ-H&N35 in patients with a feeding tube, as issues were raised regarding the response options for items related to eating and swallowing. The lack of a ‘not applicable’ response option may lead to inaccurate or missing responses in this patient subgroup. This issue should be explored further, especially as item 63 of the questionnaire assesses the use of a feeding tube, thus acknowledging that for many patients items about food consumption will not be relevant. The recall period of the questionnaires was also deemed suitable, however, the omission of a defined recall period for the first five items (forming the physical functioning scale) seems to be problematic and could introduce unwanted noise in the obtained scores [21].

This study provides a considerable volume of rich data directly elicited from patients with diversity in HNSCC locations and other clinical characteristics, and uses the input of several sources to yield a conceptual model. The study findings are therefore generalisable to patients with a similar presentation to the inclusion criteria presented in Table 1, i.e. those with a diagnosis of recurrent and/or metastatic HNSCC who have received treatment in the preceding 12 months. However, some limitations of this study are to be acknowledged. The majority of the interviewed patients were male and all were Caucasian; hence, differences in experience are only present according to these characteristics. The fact that the majority of interviewed patients had received higher education is another limitation in light of assessing item understanding; a greater number of patients who had received lower education would increase confidence in the generalisability of these findings. In addition, there was limited opportunity to understand the impacts and side effects associated with immunotherapy; only two patients had received this (as part of a clinical trial) and did not attribute any specific side effects to this treatment type.

## Conclusions

A HNSCC diagnosis and its diverse symptoms and treatments may have a negative impact on many aspects of patients’ lives. This study has identified a number of symptoms, including difficulty speaking and swallowing, localised pain, and fatigue, that may be important for treatment benefit evaluation in clinical trial from the patient’s perspective. PRO measures are an important source of data that can capture such impacts to inform patient perception of treatment effectiveness, the selection and prioritisation of endpoints during clinical trials, as well as clinical care decision making. Therefore, evidence to support the use of such measures is very important. Furthermore, reflection from the patients’ perspective can be somewhat lacking once a questionnaire is established. The findings of this study support the conceptual relevance of the EORTC QLQ-C30 and QLQ-H&N35, although additional concepts not included in the questionnaires were cited by patients. While both questionnaires were positively perceived by the majority of patients, a minority of problematic items and recall issues were identified. Thus, further research may be required to confirm the suitability of any updated or amended measure. Additional clarification is also needed when administering the questionnaires in patients with feeding tubes. The EORTC QLQ-C30 and QLQ-H&N35 are the mainstay of PRO measures for HRQoL in head and neck clinical trials; however, researchers and clinicians should be mindful of the potential downfalls of these measures, including those identified in this study. Depending on the clinical trial design, additional questionnaires may be required for higher precision in specific areas of interest but also to cover the potential shortcomings from the patient perspective identified in this qualitative study. A similar approach to the work conducted in this study may be helpful to ensure any additional questionnaires are relevant and suitable for use.

## Additional file

Additional file 1: 1. Literature review and 2. Conceptual Saturation Analysis. Literature review search strategy and list of publications finally included in the development of the patient road map. Results of the conceptual saturation analysis from the 14 patient interviews. (DOCX 84 kb)

## Abbreviations

EORTC QLQ-C30: European Organization for Research and Treatment of Cancer Quality of Life Questionnaire
EORTC QLQ-H&N35: European Organization For Research And Treatment Of Cancer Quality Of Life Questionnaire Head And Neck Module
EORTC: European Organization for Research and Treatment of Cancer
FACT-HN: Functional Assessment of Cancer Therapy-Head and Neck Subscale
FDA: Food and Drug Administration
HNSCC: Head and neck squamous cell carcinoma
HRQoL: Health-related quality of life
IRB: Independent Review Board
PRO: Patient reported outcome
PRO-CTCAE: Patient-Reported Outcomes Common Terminology Criteria for Adverse Events
Quick DASH-9: Disabilities of the Arm, Shoulder, and Hand (9-item)
UW-QOL: University of Washington Head and Neck Cancer Questionnaire
