# Supplementary material for: Patients’ experience of recurrent/metastatic head and neck squamous cell carcinoma and their perspective on the EORTC QLQ-C30 and QLQ-H&N35 questionnaires: a qualitative study
Source: J Patient Rep Outcomes. 2018 Aug 1;2:33. doi: 10.1186/s41687-018-0060-7 (PMC6092741; doi:10.1186/s41687-018-0060-7)
Supplement: Supplementary file 1 — 1. Literature review and 2. Conceptual Saturation Analysis. Literature review search strategy and list of publications finally included in the development of the patient road map. Results of the conceptual saturation analysis from the 14 patient interviews. (DOCX 84 kb) [file 41687_2018_60_MOESM1_ESM.docx]

1. Literature Review

A literature review was undertaken to identify published qualitative data in patients with Head and neck squamous cell carcinoma (HNSCC). The search strategies utilised are presented in Table S1.

Table S1: Search strategies for qualitative literature review in HNSCC

| # | MEDLINE | Results |
| --- | --- | --- |
|  | **Disease terms** |  |
| 1 | exp "Head and Neck Neoplasms"/ | 239635 |
| 2 | exp Carcinoma, Squamous Cell/ or exp Neoplasms, Squamous Cell/ | 126529 |
| 3 | 1 and 2 | 64146 |
| 4 | ("head and neck squamous cell carcinoma" or "Squamous Cell Carcinoma of the Head and Neck" or SCCHN or HNSCC).mp. [mp=title, abstract, original title, name of substance word, subject heading word, keyword heading word, protocol supplementary concept word, rare disease supplementary concept word, unique identifier] | 8866 |
| 5 | exp Mouth Neoplasms/ or exp Oropharyngeal Neoplasms/ | 58714 |
| 6 | exp Hypopharyngeal Neoplasms/ | 2354 |
| 7 | exp Laryngeal Neoplasms/ | 23838 |
| 8 | exp Salivary Gland Neoplasms/ | 13999 |
| 9 | exp Tongue Neoplasms/ | 8260 |
| 10 | exp Pharyngeal Neoplasms/ | 26024 |
| 11 | ((orophar* or hypophar* or laryn* or tongue) adj2 (cancer or carcinoma or tumo?r or neoplasm)).mp. | 12900 |
| 12 | 5 or 6 or 7 or 8 or 9 or 10 or 11 | 98359 |
| 13 | exp Carcinoma, Squamous Cell/ or exp Neoplasms, Squamous Cell/ or "squamous cell carcinoma".mp. [mp=title, abstract, original title, name of substance word, subject heading word, keyword heading word, protocol supplementary concept word, rare disease supplementary concept word, unique identifier] | 139871 |
| 14 | 12 and 13 | 30492 |
| 15 | 3 or 4 or 14 | 66999 |
|  | **Qualitative terms** |  |
| 16 | exp Qualitative Research/ | 20094 |
| 17 | focus groups/ | 16824 |
| 18 | discourse analysis.mp. | 931 |
| 19 | content analysis.mp. | 11774 |
| 20 | ethnographic research.mp. | 517 |
| 21 | ethnological research.mp. | 6 |
| 22 | constant comparative method.mp. | 898 |
| 23 | qualitative validity.mp. | 8 |
| 24 | purposive sample.mp. | 1758 |
| 25 | observational method$.mp. | 442 |
| 26 | field stud$.mp. | 10164 |
| 27 | theoretical sampl$.mp. | 346 |
| 28 | phenomenology/ | 0 |
| 29 | phenomenological research.mp. | 271 |
| 30 | cluster sampl$.mp. | 3993 |
| 31 | ethnonursing.af. | 88 |
| 32 | ethnograph$.mp. | 6035 |
| 33 | phenomenol$.af. | 15981 |
| 34 | grounded theory.mp. | 5966 |
| 35 | (grounded adj (theor$ or study or studies or research or analys?s)).af. | 6060 |
| 36 | (emic or etic or hermeneutic$ or heuristic$ or semiotic$).af. or (data adj1 saturat$).tw. or participant observ$.tw. | 13665 |
| 37 | (action research or cooperative inquir$ or co operative inquir$ or co-operative inquir$).mp. | 2464 |
| 38 | (humanistic or existential or experiential or paradigm$).mp. | 96518 |
| 39 | human science.tw. | 210 |
| 40 | biographical method.tw. | 12 |
| 41 | qualitative validity.af. | 8 |
| 42 | purposive sampl$.af. | 3066 |
| 43 | theoretical sampl$.af. | 346 |
| 44 | ((purpos$ adj4 sampl$) or (focus adj group$)).af. | 32200 |
| 45 | (life world or life-world or conversation analys?s or personal experience$ or theoretical saturation).mp. | 11474 |
| 46 | lived experience$.tw. | 2407 |
| 47 | life experience$.mp. | 3047 |
| 48 | cluster sampl$.mp. | 3993 |
| 49 | (theme$ or thematic).mp. | 48121 |
| 50 | categor$.mp. | 213177 |
| 51 | observational method$.af. | 442 |
| 52 | field stud$.mp. | 10164 |
| 53 | focus group$.af. | 26478 |
| 54 | questionnaire$.mp. | 460539 |
| 55 | content analysis.af. | 11774 |
| 56 | thematic analysis.af. | 4298 |
| 57 | constant comparative.af. | 1639 |
| 58 | discourse analys?s.af. | 962 |
| 59 | ((discourse$ or discurs$) adj3 analys?s).tw. | 1227 |
| 60 | (constant adj (comparative or comparison)).af. | 2358 |
| 61 | narrative analys?s.af. | 565 |
| 62 | heidegger$.tw. | 486 |
| 63 | colaizzi$.tw. | 382 |
| 64 | speigelberg$.tw. | 1 |
| 65 | (van adj manen$).tw. | 218 |
| 66 | (van adj kaam$).tw. | 25 |
| 67 | (merleau adj ponty$).tw. | 126 |
| 68 | husserl$.tw. | 162 |
| 69 | giorgi$.tw. | 381 |
| 70 | foucault$.tw. | 552 |
| 71 | (corbin$ adj2 strauss$).tw. | 156 |
| 72 | (strauss$ adj2 corbin$).tw. | 156 |
| 73 | (glaser$ adj2 strauss$).tw. | 81 |
| 74 | glaser$.tw. | 735 |
| 75 | findings.af. | 1324653 |
| 76 | interview$.af. or Interviews/ | 250100 |
| 77 | qualitative.af. | 132041 |
| 78 | 16 or 17 or 18 or 19 or 20 or 21 or 22 or 23 or 24 or 25 or 26 or 27 or 28 or 29 or 30 or 31 or 32 or 33 or 34 or 35 or 36 or 37 or 38 or 39 or 40 or 41 or 42 or 43 or 44 or 45 or 46 or 47 or 48 or 49 or 50 or 51 or 52 or 53 or 54 or 55 or 56 or 57 or 58 or 59 or 60 or 61 or 62 or 63 or 64 or 65 or 66 or 67 or 68 or 69 or 70 or 71 or 72 or 73 or 74 or 75 or 76 or 77 or 78 | 2255488 |
| 79 | 15 and 78 | 7820 |
| 80 | limit 79 to yr="2000 -Current" | 5334 |
| 81 | limit 80 to "all adult (19 plus years)" | 3559 |
| 82 | limit 81 to humans | 3559 |
| 83 | limit 82 to english language | 3320 |
| **#** | **EMBASE** | **Results** |
|  | **Disease terms** |  |
| 1 | exp "Head and Neck Neoplasms"/ | 218144 |
| 2 | exp Carcinoma, Squamous Cell/ or exp Neoplasms, Squamous Cell/ | 96781 |
| 3 | 1 and 2 | 32768 |
| 4 | ("head and neck squamous cell carcinoma" or "Squamous Cell Carcinoma of the Head and Neck" or SCCHN or HNSCC).mp. [mp=title, abstract, subject headings, heading word, drug trade name, original title, device manufacturer, drug manufacturer, device trade name, keyword] | 12180 |
| 5 | exp Mouth Neoplasms/ or exp Oropharyngeal Neoplasms/ | 80121 |
| 6 | exp Hypopharyngeal Neoplasms/ | 4072 |
| 7 | exp Laryngeal Neoplasms/ | 26868 |
| 8 | exp Salivary Gland Neoplasms/ | 15277 |
| 9 | exp Tongue Neoplasms/ | 8826 |
| 10 | exp Pharyngeal Neoplasms/ | 23655 |
| 11 | ((orophar* or hypophar* or laryn* or tongue) adj2 (cancer or carcinoma or tumo?r or neoplasm)).mp. | 45071 |
| 12 | 5 or 6 or 7 or 8 or 9 or 10 or 11 | 104888 |
| 13 | exp Carcinoma, Squamous Cell/ or exp Neoplasms, Squamous Cell/ or "squamous cell carcinoma".mp. [mp=title, abstract, subject headings, heading word, drug trade name, original title, device manufacturer, drug manufacturer, device trade name, keyword] | 123578 |
| 14 | 12 and 13 | 27034 |
| 15 | 3 or 4 or 14 | 46050 |
|  | **Qualitative Research** |  |
| 16 | exp Qualitative Research/ | 25429 |
| 17 | focus groups/ | 159933 |
| 18 | discourse analysis.mp. | 1477 |
| 19 | content analysis.mp. | 14721 |
| 20 | ethnographic research.mp. | 938 |
| 21 | ethnological research.mp. | 8 |
| 22 | constant comparative method.mp. | 1011 |
| 23 | qualitative validity.mp. | 114 |
| 24 | purposive sample.mp. | 2121 |
| 25 | observational method$.mp. | 1504 |
| 26 | field stud$.mp. | 12623 |
| 27 | theoretical sampl$.mp. | 471 |
| 28 | phenomenology/ | 6871 |
| 29 | phenomenological research.mp. | 308 |
| 30 | cluster sampl$.mp. | 4709 |
| 31 | ethnonursing.af. | 94 |
| 32 | ethnograph$.mp. | 7188 |
| 33 | phenomenol$.af. | 20054 |
| 34 | grounded theory.mp. | 7174 |
| 35 | (grounded adj (theor$ or study or studies or research or analys?s)).af. | 7290 |
| 36 | (emic or etic or hermeneutic$ or heuristic$ or semiotic$).af. or (data adj1 saturat$).tw. or participant observ$.tw. | 15604 |
| 37 | (action research or cooperative inquir$ or co operative inquir$ or co-operative inquir$).mp. | 3024 |
| 38 | (humanistic or existential or experiential or paradigm$).mp. | 112594 |
| 39 | human science.tw. | 245 |
| 40 | biographical method.tw. | 16 |
| 41 | qualitative validity.af. | 114 |
| 42 | purposive sampl$.af. | 3770 |
| 43 | theoretical sampl$.af. | 471 |
| 44 | ((purpos$ adj4 sampl$) or (focus adj group$)).af. | 33813 |
| 45 | (life world or life-world or conversation analys?s or personal experience$ or theoretical saturation).mp. | 24058 |
| 46 | lived experience$.tw. | 2734 |
| 47 | life experience$.mp. | 3859 |
| 48 | cluster sampl$.mp. | 4709 |
| 49 | (theme$ or thematic).mp. | 58745 |
| 50 | categor$.mp. | 272890 |
| 51 | observational method$.af. | 1504 |
| 52 | field stud$.mp. | 12623 |
| 53 | focus group$.af. | 26638 |
| 54 | questionnaire$.mp. | 529514 |
| 55 | content analysis.af. | 14721 |
| 56 | thematic analysis.af. | 5981 |
| 57 | constant comparative.af. | 1820 |
| 58 | discourse analys?s.af. | 1508 |
| 59 | ((discourse$ or discurs$) adj3 analys?s).tw. | 1405 |
| 60 | (constant adj (comparative or comparison)).af. | 2701 |
| 61 | narrative analys?s.af. | 642 |
| 62 | heidegger$.tw. | 544 |
| 63 | colaizzi$.tw. | 419 |
| 64 | speigelberg$.tw. | 0 |
| 65 | (van adj manen$).tw. | 238 |
| 66 | (van adj kaam$).tw. | 27 |
| 67 | (merleau adj ponty$).tw. | 138 |
| 68 | husserl$.tw. | 205 |
| 69 | giorgi$.tw. | 466 |
| 70 | foucault$.tw. | 592 |
| 71 | (corbin$ adj2 strauss$).tw. | 177 |
| 72 | (strauss$ adj2 corbin$).tw. | 177 |
| 73 | (glaser$ adj2 strauss$).tw. | 96 |
| 74 | glaser$.tw. | 694 |
| 75 | findings.af. | 1494357 |
| 76 | interview$.af. or Interviews/ | 290104 |
| 77 | qualitative.af. | 164041 |
| 78 | 16 or 17 or 18 or 19 or 20 or 21 or 22 or 23 or 24 or 25 or 26 or 27 or 28 or 29 or 30 or 31 or 32 or 33 or 34 or 35 or 36 or 37 or 38 or 39 or 40 or 41 or 42 or 43 or 44 or 45 or 46 or 47 or 48 or 49 or 50 or 51 or 52 or 53 or 54 or 55 or 56 or 57 or 58 or 59 or 60 or 61 or 62 or 63 or 64 or 65 or 66 or 67 or 68 or 69 or 70 or 71 or 72 or 73 or 74 or 75 or 76 or 77 | 2735227 |
| 79 | 15 and 78 | 5410 |
| 80 | limit 79 to yr="2000 -Current" | 4356 |
| 81 | limit 80 to adult <18 to 64 years> | 1907 |
| 82 | limit 81 to human | 1906 |
| 83 | limit 82 to english language | 1797 |

From these searches a total of 25 publications were finally included in the development of the patient road map, as follows:

1. Andreassen S, Randers I, Naslund E, Stockeld D, Mattiasson AC. Patients' experiences of living with oesophageal cancer. Journal of clinical nursing. 2006 Jun;15(6):685-95.

2. Callahan C. Facial Disfigurement and Sense of Self in Head and Neck Cancer. US: Haworth Press; 2004. p. 73-87.

3. Charalambous AC, M.; Tsitsi, T. Narratives of patients living with radiation-induced xerostomia: Demythologizing the symptom. Supportive Care in Cancer. 2014 June;1):S224.

4. Chen S-C. Life experiences of Taiwanese oral cancer patients during the postoperative period. Scandinavian Journal of Caring Sciences. 2012;26(1):98-103.

5. Fletcher BSC, Marlene Z.; Schumacher, Karen; Lydiatt, William. A blessing and a curse: Head and neck cancer survivors' experiences. Cancer Nursing. 2012;35(2):126-32.

6. Goldstein NEG, Eric; Morrison, R. Sean. Palliative care for patients with head and neck cancer: "I would like a quick return to a normal lifestyle.". US: American Medical Assn; 2008. p. 1818-25.

7. Johansson MR, Anna; Ahlberg, Karin; Finizia, Caterina. “Setting boundaries” – Mental adjustment to cancer in laryngeal cancer patients: An interview study. European Journal of Oncology Nursing. 2012;16(4):419-25.

8. Konradsen HK, Marit; McCallin, Antoinette; Cayé-Thomasen, Per; Zoffmann, Vibeke. Breaking the silence: Integration of facial disfigurement after surgical treatment for cancer. Qualitative Health Research. 2012;22(8):1037-46.

9. Larsson MH, Birgitta; Athlin, Elsy. Needing a hand to hold: Lived experiences during the trajectory of care for patients with head and neck cancer treated with radiotherapy. Cancer Nursing. 2007;30(4):324-34.

10. Loewen IJB, Carol A.; Seikaly, Hadi; Harris, Jeffrey; Rieger, Jana M. Quality of life in patients with hemiglossectomy: Comparison of the EORTC QLQ-H&N35 and a semi-structured interview. Canadian Journal of Speech-Language Pathology and Audiology. 2009;33(4):189-95.

11. McLane LJ, Katherine; Lydiatt, William; Lydiatt, Daniel; Richards, Alan. Taking away the fear: A grounded theory study of cooperative care in the treatment of head and neck cancer. Psycho-Oncology. 2003;12(5):474-90.

12. Missel M, Birkelund R. Living with incurable oesophageal cancer. A phenomenological hermeneutical interpretation of patient stories. European journal of oncology nursing : the official journal of European Oncology Nursing Society. 2011 Sep;15(4):296-301.

13. Moore KAF, P. J.; Farah, C. S. “I have quality of life…but…”: Exploring support needs important to quality of life in head and neck cancer. European Journal of Oncology Nursing. 2014;18(2):192-200.

14. Moore RJC, R. M.; Khuri, F. R. Communicating suffering in primary stage head and neck cancer. European Journal of Cancer Care. 2004;13(1):53-64.

15. Nund RL, Ward EC, Scarinci NA, Cartmill B, Kuipers P, Porceddu SV. Survivors' experiences of dysphagia-related services following head and neck cancer: implications for clinical practice. International journal of language & communication disorders / Royal College of Speech & Language Therapists. 2014 May-Jun;49(3):354-63.

16. O’Brien KR, Brenda; Low, Christopher; Deyn, Lorraine; Rogers, Simon N. An exploration of the perceived changes in intimacy of patients’ relationships following head and neck cancer. Journal of Clinical Nursing. 2012;21(17-18):2499-508.

17. Ottosson SL, Göran; Olsson, Cecilia. The experience of food, eating and meals following radiotherapy for head and neck cancer: A qualitative study. Journal of Clinical Nursing. 2013;22(7-8):1034-43.

18. Penner JLM, Susan; Lobchuk, Michelle; Daeninck, Paul. Family members' experiences caring for patients with advanced head and neck cancer receiving tube feeding: A descriptive phenomenological study. Journal of Pain and Symptom Management. 2012;44(4):563-71.

19. Rose P, Yates P. Quality of life experienced by patients receiving radiation treatment for cancers of the head and neck. Cancer Nurs. 2001 Aug;24(4):255-63.

20. Scott SEG, E. A.; Main, J.; McGurk, M. Patient delay in oral cancer: a qualitative study of patients' experiences. Psycho-Oncology. 2006;15(6):474-85.

21. Semple CJD, Lynn; Kernohan, William George; McCaughan, Eilis; Sullivan, Kate. Changes and challenges to patients' lifestyle patterns following treatment for head and neck cancer. Journal of Advanced Nursing. 2008;63(1):85-93.

22. Semple CJM, Tanya. Experience of parents with head and neck cancer who are caring for young children. Journal of Advanced Nursing. 2010;66(6):1280-90.

23. Thambyrajah CH, Jim; Altman, Keith; Llewellyn, Carrie. “Cancer doesn't mean curtains”: Benefit finding in patients with head and neck cancer in remission. Journal of Psychosocial Oncology. 2010;28(6):666-82.

24. Tong MCFL, K. Y. S.; Yuen, M. T. Y.; Lo, P. S. Y. Perceptions and experiences of post‐irradiation swallowing difficulties in nasopharyngeal cancer survivors. European Journal of Cancer Care. 2011;20(2):170-8.

25. Turpin MD, Rudi; Owen, Ray; Thomas, Mike. The meaning and impact of head and neck cancer: An interpretative phenomenological and repertory grid analysis. Journal of Constructivist Psychology. 2009;22(1):24-54.

1. Example questions from the interview guide

Some of the questions within the interview guide can be seen below in Table S2.

| **Example concept elicitation questions (related probe questions were also included)** |
| --- |
| - So, to start with, can you tell me about your experience of [location of tumor] cancer, let’s start when you were first diagnosed, how did your diagnosis come about? - Can you tell me about the time between diagnosis and the starting your first treatment? - Can you tell what it is/was like having treatment(s) for your [location of tumor] cancer? - Thinking back to the [symptoms mentioned in response to Q1] you mentioned happening before you were treated, can you tell me how these are now? - So finally, we’re also interested in understanding any support you’ve had during your recovery. Could you talk to me about any experiences of this? |
| **Example concept elicitation questions (Each questionnaire was debriefed on an item by item basis, in addition to general questions)** |
| Instruction questions:   - In your own words, what is this instruction telling you to do? - How could this instruction be worded more clearly? - How could the layout or format of this instruction be improved?   Questions asked for each item of each questionnaire:   - In your own words, what does the question mean? - How would you suggest rewording this question to make it clearer? - What do you think about the choices you were given for your answer? - You answered X to this question, why was that? What were you thinking about? - How far back were you thinking when you answered this question?   Overall questions:   - Overall what did you think about the questionnaire? - How easy or difficult was it to complete? - Is there anything important you think is missing from the questionnaire? - Were there any questions you had difficulty with or didn’t want to answer? Which ones? |

1. Conceptual Saturation Analysis

Conceptual saturation analysis was conducted and results are presented in Table S3.

Results for the 14 patient interviews are presented.

Overall, it was determined that conceptual saturation was met based on the 14 interviews; almost all concepts arose in the first set of interviews and in-depth, rich data was obtained which provided a comprehensive qualitative insight into HNSCC patient experience.

Interestingly, only two new impacts ‘difficulty walking’ and ‘feeling guilty’ arose in the second set (last two interviews), compared with eight new symptoms/side effects:

- Constipation (n=1 patient)
- Fever (n=1 patient)
- Hearing problems (n=1 patient)
- Impotence (n= 1 patient)
- Tongue ulcer (n= 1 patient)
- Sticky saliva (n=2 patients)
- Swelling/ fluid retention (n=3 patients)
- Oral inflammation/ burning sensation (n=1)

One explanation for these additional concepts arising in the second set may be that due to the variability in tumour location. Although all patients were diagnosed with HNSCC, the specific type of tumour varied (oral, larynx, oropharynx, hypopharynx, nasopharynx) which may explain why individual patients experienced quite different and specific symptoms and side effects. As these concepts were each mentioned by a small number of patients (n=6), it was deemed that they were maybe related to individual experience and circumstance, and therefore not a sign that conceptual saturation had not been reached.

There were also three initial symptoms of the disease that were described by clinicians that were not reported during the patient interviews: difficulty chewing food, enlarged tonsils, and headaches. These have been added to the saturation table and highlighted accordingly.

**Table S3: Conceptual saturation analysis**

|  | Set 1 | | | | | |  | Set 2 | | | | | | | |  | Comments |
| --- | --- | --- | --- | --- | --- | --- | --- | --- | --- | --- | --- | --- | --- | --- | --- | --- | --- |
| Patient ID | 01-01-M-83 | 01-03-M-55 | 01-04 M-59 | 01-06 M-57 | 01-09 M-66 | 01-10 M-35 |  | 01-11-M-62 | 01-05-M-57 | 01-07 F-66 | 01-08-M-82 | 01-12-M-70 | 01-13 F-62 | 01-14-F-61 | 01-15-F-32 |  |  |
| **Symptoms and side effects** | | | | | | | | | | | | | | | | |  |
| **Abdominal bloating** |  |  |  |  |  | X | 1 |  |  |  |  |  |  |  |  | 0 |  |
| **Bleeding** | X |  | X |  |  |  | 2 |  |  |  |  |  |  |  |  | 0 | Mentioned by patients as a treatment side effect, but also by clinicians as an initial symptom (specifically nasal bleeding) |
| **Breathing problems** | X |  |  |  |  | X | 2 |  |  |  |  |  |  |  | x | 1 | Mentioned by patients as a treatment side effect, but also by clinicians as an initial symptom |
| **Change in voice** |  |  |  |  | X |  | 1 |  |  |  |  |  |  |  |  | 0 |  |
| **Constipation** |  |  |  |  |  |  | 0 |  |  | X |  |  |  |  |  | 1 | Arose in final set only |
| **Cough** | X |  |  |  | X |  | 2 |  |  |  |  |  |  |  |  | 0 |  |
| **Diarrhoea** |  |  |  |  |  | X | 1 |  |  |  |  |  |  |  |  | 0 |  |
| **Difficulty speaking/slurred speech** | X |  |  | X |  | X | 3 |  |  | X |  | X | X | X | x | 5 | Mentioned by patients as a treatment side effect, but also by clinicians as an initial symptom |
| **Difficulty chewing food** |  |  |  |  |  |  |  |  |  |  |  |  |  |  |  |  | Concept mentioned by clinicians as a potential symptom |
| **Dizziness** |  |  |  |  |  | X | 1 |  |  |  |  |  |  |  |  | 0 |  |
| **Difficulty sleeping** |  |  | X |  |  |  | 1 |  |  |  |  |  |  |  | x | 1 |  |
| **Dry mouth/lack of saliva** |  | X | X | X |  |  | 3 |  | X | X | X | X |  |  |  | 4 |  |
| **Dry/peeling skin on the neck** |  |  |  |  | X | X | 2 |  |  | X |  | X |  |  |  | 2 |  |
| **Ear pain** |  |  |  |  |  |  |  |  |  |  |  |  |  |  |  |  | Concept mentioned by clinicians as a potential symptom |
| **Enlarged tonsils** |  |  |  |  |  |  |  |  |  |  |  |  |  |  |  |  | Concept did not arise from patient interviews specifically, although likely would have been reported by patients as swelling, or sore throat. Mentioned by clinicians as a potential symptom |
| **Excessive mucous in the mouth** |  | X | X |  |  |  | 2 |  |  |  |  |  |  |  |  | 0 |  |
| **Fever** |  |  |  |  |  |  | 0 |  | X |  |  |  |  |  |  | 1 | Arose in final set only |
| **Hair loss** |  |  |  |  | X |  | 1 |  |  |  |  | X | X |  | X | 3 |  |
| **Hearing problems** |  |  |  |  |  |  | 0 |  |  |  |  |  |  | X |  | 1 | Arose in final set only  Mentioned by patients as a treatment side effect, but also by clinicians as an initial symptom |
| **Headaches** |  |  |  |  |  |  |  |  |  |  |  |  |  |  |  |  | Concept mentioned by clinicians as a potential symptom |
| **Impact on range of movement** | X |  |  |  |  | X | 2 |  |  |  |  | X |  |  |  | 1 |  |
| **Impotence** |  |  |  |  |  |  | 0 |  |  |  |  | X |  |  |  | 1 | Arose in final set only |
| **Loss of appetite/eating less** | X |  |  |  |  | X | 2 |  |  |  |  |  |  |  |  | 0 |  |
| **Loss of voice** | X | X |  |  |  |  | 2 |  |  |  |  |  |  |  |  | 0 |  |
| **Lump/bumps; head or neck** | X |  |  | X |  | X | 3 | X | X |  | X |  |  | X | X | 5 |  |
| **Nausea** |  | X |  |  |  | X | 2 |  |  | X |  |  | X | X | X | 4 |  |
| **Oral inflammation/ burning sensation** |  |  |  |  |  |  |  |  |  |  |  |  |  |  | X | 1 | Arose in final set only |
| **Peripheral neuropathy** |  |  | X |  |  |  | 1 |  |  |  |  |  |  |  |  | 0 |  |
| **Numbness** |  |  |  | X |  | X | 2 | X | X | X |  |  |  |  |  | 3 |  |
| **Pain** | X |  | X | X |  | X | 4 |  | X | X | X | X |  | X | X | 6 |  |
| **Psychedelic dreams** |  |  |  |  | X |  | 1 |  |  |  |  |  |  |  |  | 0 |  |
| **Radiation burns** | X | X | X |  |  |  | 3 |  |  |  |  | X |  | X | X | 3 |  |
| **Soreness/Discomfort** | X |  | X | X | X |  | 4 | X |  | X |  | X |  |  |  | 3 |  |
| **Sticky saliva** |  |  |  |  |  |  | 0 |  | X |  |  | X |  |  |  | 2 | Arose in final set only |
| **Swallowing problems** |  | X | X | X | X |  | 4 | X | X |  | X | X |  | X | X | 6 |  |
| **Swelling/fluid retention** |  |  |  |  |  |  | 0 | X |  | X |  |  |  |  | X | 3 | Arose in final set only |
| **Taste problems** |  | X |  | X | X |  | 3 |  | X | X | X | X |  |  |  | 4 |  |
| **Tiredness/fatigue/exhaustion** | X | X | X | X |  | X | 5 | X |  |  |  | X | X | X | X | 5 |  |
| **Tongue ulcer** |  |  |  |  |  |  | 0 |  |  |  |  |  |  | X |  | 1 | Arose in final set only |
| **Tooth damage/removal** |  |  | X |  |  |  | 1 |  |  | X |  | X |  |  |  | 2 |  |
| **Vomiting** |  |  |  |  |  | X | 1 |  |  |  |  |  | X | X |  | 2 |  |
| **Weakness** |  |  |  | X |  | X | 2 |  | X |  |  |  |  | X | X | 3 |  |
| **Weight loss** | X | X | X |  |  |  | 3 |  | X | X | X | X | X | X | X | 7 |  |
| **Emotional/psychological impacts** | | | | | | | | | | | | | | | | | |
| **Bothered/embarrassed by appearance** |  |  |  | X |  |  | 1 |  |  | X |  | X |  | X | X | 4 |  |
| **Depression/demotivation/ feeling hopeless** |  | X |  | X |  |  | 2 |  | X |  |  |  |  | X | X | 3 |  |
| **Fear/worry/concern** |  | X |  | X | X | X | 4 | X | X | X | X | X |  | X | X | 7 |  |
| **Feeling drained/worn down** |  |  |  | X |  |  | 1 |  |  |  |  |  |  |  |  | 0 |  |
| **Feeling irritated/annoyed/frustrated** | X |  |  | X |  |  | 2 |  |  |  |  | X |  | X |  | 2 |  |
| **Feeling isolated** |  | X |  |  |  |  | 1 |  |  |  |  |  |  |  | X | 1 |  |
| **Feeling overwhelmed** |  | X |  |  |  |  | 1 |  |  | X |  |  |  |  | X | 2 |  |
| **Feeling guilty** |  |  |  |  |  |  |  |  |  |  |  |  |  |  | X | 1 | Arose in final set only |
| **Missing/not enjoying food/feeling hungry** | X | X |  | X |  |  | 3 |  |  | X |  | X |  |  | X | 3 |  |
| **Shock/surprise/disbelief of diagnosis** |  | X |  | X | X |  | 3 |  |  |  |  | X |  |  |  | 1 |  |
| **Upset/miserable** |  |  |  |  |  | X | 1 | X |  |  |  | X |  |  |  | 2 |  |
| **Physical and daily activities impacts** | | | | | | | | | | | | | | | | | |
| **Cannot drive** | X |  | X |  |  | X | 3 |  |  |  |  |  | X | X | X | 3 |  |
| **Difficulty eating** | X |  |  | X | X |  | 3 |  | X | X | X | X | X | X | X | 7 |  |
| **Difficulty carrying items** |  |  |  |  |  | X | 1 |  |  |  |  |  |  | X |  | 1 |  |
| **Difficulty drinking** |  |  | X | X |  |  | 2 |  | X | X | X |  | X |  |  | 4 |  |
| **Difficulty sleeping; e.g. from pain /coughing/excessive mucous/after surgery** | X | X | X |  | X |  | 4 |  |  | X |  |  | X | X |  | 4 |  |
| **Difficulty standing** |  |  | X |  |  |  | 1 |  |  |  |  |  |  |  |  | 0 |  |
| **Difficulty walking** |  |  |  |  |  |  | 0 |  |  |  |  |  |  | 1 | 1 | 2 | Arose in final set only |
| **Difficulty with self-care** |  |  |  | X |  | X | 2 | X | X |  |  |  |  | X | X | 4 |  |
| **Increased day napping/sleeping** |  | X |  | X |  |  | 2 |  |  |  |  | X |  |  |  | 1 |  |
| **Work and financial impacts** | | | | | | | | | | | | | | | | | |
| **Cannot work/reduced ability to work** |  |  | X | X |  | X | 3 |  | X | X |  |  | X |  | X | 4 |  |
| **Disease is a financial burden** |  |  |  |  |  | X | 1 |  |  | X |  |  |  |  |  | 1 |  |
| **Socialising/relationships/lifestyle/hobby impacts:** | | | | | | | | | | | | | | | | | |
| **Concern of disease impact on family** |  | X |  |  |  |  | 1 |  | X |  |  |  |  |  |  | 1 |  |
| **Difficulties with intimacy** |  |  |  |  |  |  |  |  |  |  |  |  |  |  |  |  | Concept mentioned by clinicians as a potential impact |
| **Reliance on partner/others** | X |  |  |  |  |  | 1 |  |  |  |  |  | X | X | X | 3 |  |
| **Unable/limited to continue with socialising/hobbies** |  | X | X | X | X |  | 4 |  | X |  | X | X | X |  | X | 4 |  |
